# Improving Coverage Rate for Urban Link Travel Time Prediction Using Probe Data in the Low Penetration Rate Environment

**DOI:** 10.3390/s20010265

**Published:** 2020-01-02

**Authors:** Ruotian Tang, Ryo Kanamori, Toshiyuki Yamamoto

**Affiliations:** 1 Department of Civil Engineering, Nagoya University, Nagoya, Aichi 464-8603, Japan; 2 Institute of Innovation for Future Society, Nagoya University, Nagoya, Aichi 464-8603, Japan; kanamori.ryo@nagoya-u.jp; 3 Institute of Materials and Systems for Sustainability, Nagoya University, Nagoya, Aichi 464-8603, Japan; yamamoto@civil.nagoya-u.ac.jp

**Keywords:** urban travel time prediction, low penetration rate, coverage rate, vehicles in the crossing direction, short-term, probe vehicle data

## Abstract

Short-term travel time prediction is an important consideration in modern traffic control and management systems. As probe data technology has developed, research interest has moved from highways to urban roads. Most research has only focused on improving the prediction accuracy on urban roads because it is the key index of evaluating a model. However, the low penetration rate of probe vehicles at urban networks may result in the low coverage rate which restricts prediction models from practical applications. This research proposed a non-parametric model based on Bayes’ theorem and a resampling process to predict short-term urban link travel time, which can enhance the coverage rate while maintaining the prediction accuracy. The proposed model used data from vehicles in both the target link and its crossing direction, so its coverage rate can be expanded, especially when the data penetration rate is low. In addition, the utilization of relationships between vehicles in both directions can reflect the influence of signal timing. The proposed model was evaluated in a computer simulation to test its robustness and reliability under different data penetration rates. The results implied that the proposed model has a high coverage rate, demonstrating stable and acceptable performance at different penetration rates.

## 1. Introduction

It is undoubtable that advanced traffic control and management systems, such as intelligent transportation systems (ITS), are indispensable tools for modern cities. As living standards improve, the public is no longer satisfied with a transportation system that can only deal with traffic problems that have already happened. A timely, reliable, and safe transportation system that can predict traffic conditions is hence required [1]. As a crucial part of such transportation systems, short-term traffic forecasting, which makes predictions from several seconds to several hours into the future, has attracted much research interest and effort for more than three decades [2]. Among all types of traffic information, travelers usually make decisions based on travel time because it is easier to understand than other types of traffic information. Many researchers have focused on the route travel time, but Shi et al. [3] argued that the route travel time can be formulated as the sum of the link travel times. Using this formulation, the distinct travel time delays due to traffic signals and different turning behaviors can be well captured. It is also more flexible to use the link travel time because it is difficult to predict the travel demand and the routes that travelers may take. Elhenawy et al. [4] pointed out that for long trips, traffic conditions might change significantly so travel time prediction is preferred to provide dynamic information for the entire trip. Therefore, this study concentrated on predicting the link travel time rather than the route travel time.

To predict the travel time, data are collected from two main sources: the fixed traffic sensor and the probe vehicle [3]. Fixed traffic sensors, which include loop detectors and traffic monitors, are installed at specific locations of the highway and urban road. Such sensors can directly collect traffic information like traffic volume and travel speed, and travel time can be deduced from them. However, the relation between travel time and other traffic information differs under different traffic conditions and study sites, so it is difficult to propose a general relation for wide application. In addition, the installation and maintenance cost of fixed sensors is high, and they cannot cover each corner of the city. With the development of data collection technology, probe vehicles are drawing more and more attention because of their relatively low cost and wider coverage. These specific vehicles (e.g., taxis and buses) are equipped with onboard GPS units to collect real-time travel time information for the whole urban network, including areas without fixed traffic sensors. Therefore, more and more researchers tend to predict travel time with data from probe vehicles instead of fixed traffic sensors for real-time traffic control and management, especially on urban networks.

For research which utilizes probe data, the amount of data collected relies on two variables: the penetration rate of probe vehicles and their frequency [5]. The data frequency can be increased easily as long as the computer is able to handle and store these data, whereas it is difficult to increase the penetration rate because the public is reluctant to change their vehicles into probe vehicles. Lu et al. [6] pointed out that a high frequency requires the penetration rate at a relatively high level, so the influence of penetration rate on the short-term travel time prediction should be fully considered. Most approaches to short-term prediction using probe data examined freeways or urban arterials [7,8,9], but they did not consider the low penetration rate because traffic on freeways or urban arterials is relatively high. Argote-Cabañero et al. [10] emphasized that higher traffic results in higher variability so it might be an increased need for higher penetration rates. On the other hand, there is an increasing motivation to expand the forecasting scale to urban networks where the penetration rate is low. Even though each vehicle might be equipped with onboard GPS units in the future, most researchers agreed that the penetration rate of vehicles sending probe data to the transportation system is still limited due to the privacy issue, and the cost for data processing and storage [11,12,13,14,15,16,17]. Therefore, a part of the literature has focused on determining the optimal penetration rate for providing accurate traffic information [6,13,16]. To obtain reliable traffic information, the optimal penetration rate ranges from 1% to 60% because it depends on network characteristics and estimation methods. Bellavista et al. [12] argued that it is impractical that probe vehicles are assumed to be distributed uniformly on the network in most researches. Even though there might be an agreement on the best penetration rate of probe vehicles, it seems far from real cases of practical application.

Srinivasan and Jovanis [16] implied that the penetration rate influences both coverage rate and accuracy of travel time measurement on the urban network, but most researches only focused on the accuracy. Although many developed models can make quite accurate predictions at study sites where the penetration rate is high, it is doubtable that they have a high coverage rate for the practical application when the penetration rate is low. In this study, the coverage rate is defined as the proportion of targets a model can predict. The main objective of this study is to enhance the coverage rate and maintain accuracy at the same time when predicting the link travel time on urban networks in the low penetration rate environment. To realize the objective, this study proposed a non-parametric model which not only utilized the spatiotemporal relationship between vehicles in the target link, but also took advantage of the spatiotemporal relationship between vehicles in the target link and vehicles in the crossing direction. To demonstrate the proposed model can achieve an acceptable prediction accuracy, two conventional non-parametric models based on the k-nearest neighbors (kNN) and the particle filtering (PF) approaches, were used for comparison. Since current real-world probe data are usually under low penetration rate, researchers often used the computer simulation to evaluate the performance of the model and the influence of different penetration rates of probe vehicles [5,18]. For the same reason, in this study, a computer simulation was employed to compare the performance of the three models at different penetration rates.

The rest of this paper is organized as follows. Section 2 introduces researches which used to improve the accuracy of travel time estimation when the penetration rate is low and researches concerning travel time prediction. Section 3 explains the details of the proposed model. Section 4 describes the simulation datasets used in this study. In Section 5, the performance of the proposed model is analyzed and compared with other models under different penetration rates. The last section provides the conclusions and future work of this study.

## 2. Related Work

So far, many researchers have devoted their efforts to improving the estimation of travel time when the penetration rate is low. For example, Wan et al. [17] reconstructed the maximum likelihood trajectory to estimate the time spent on each segment of the road; Li et al. [19] reconstructed vehicle trajectories based on a kNN regression algorithm so as to support travel time estimation; Jenelius and Koutsopoulos [14] proposed a statistical regression model to estimate urban road travel time by vehicle trajectory data and included correlation between travel times of different links based on a spatial moving average structure to capture the spatiotemporal variations in speeds; Sanaullah et al. [15] used a distance and time proportion method based on the map-matched points from adjacent links to enhance the coverage rate and to reduce the uncertainty of the estimation, and further applied spatial and temporal moving average to improve the accuracy; Alrukaibi et al. [11] used simulation to increase the sample size and employed neighbor links to estimate the travel time of the target link. However, there are few researchers who consider problems resultant from the low penetration rate when making travel time prediction because most study sites for travel time prediction were highways and urban arterials where data are easy to collect.

As an important part of the ITS, travel time prediction has attracted research interest for a long time. Mori et al. [20] reviewed models that have been developed for travel time prediction and categorized them into four types: naïve model, traffic flow-based model, data-based model, and hybrid model. Naïve and traffic flow-based models usually make restrictive assumptions which have difficulty to describe general situations in real cases. Data based models have advantages over naïve and traffic flow-based model because there is no requirement for specific theories and assumptions, but abundant data are needed. Hybrid models attempt to combine different models regardless of their types to make a more accurate prediction. Since the application of probe vehicles makes large amounts of data available, data-based models are drawing increasing attention. Data based models can be further grouped into parametric models (e.g., linear regression, Bayesian net, and time-series models) and non-parametric models (e.g., neural networks (NN), pattern recognition and support vector machine models). Under complicated traffic situations such as urban networks, non-parametric models outperform parametric models and it is relatively easy for non-parametric models to be extended from one application to another [2,21,22]. However, the influence of penetration rate is more significant for non-parametric models, such as the kNN-based model [9,21,23] and the PF-based model [7,24], because these models require time-sequential samples that cover exactly the same time interval and have exactly the same sample length.

It is widely recognized that traffic of roads on the same network often affects each other, especially those on adjacent links. For example, Bauer et al. [25] investigated the travel time covariance between pairs of urban links when making the travel time prediction and found out that there were significant correlations between residuals on adjacent links. They pointed out it is necessary to add the travel time covariance when summing up the predicted link travel time and its prediction error to generate the predicted route travel time. Therefore, spatiotemporal relationships were often added to different types of models to improve accuracy when predicting travel time and other traffic-related parameters. Wang et al. [26] reviewed traditional statistical models which included space-time autoregressive integrated moving average (STARIMA) and proposed a space-time delay neural network model (STDNN) which integrated the spatiotemporal autocorrelation of road traffic networks using NN. They used data collected from automatic number plate recognition cameras in London for travel time prediction and showed that the STDNN is more accurate than the STARIMA because it can capture spatiotemporal autocorrelation locally and dynamically. Cai et al. [21] applied a two-dimensional spatiotemporal state matrix to improve the accuracy of the kNN when making multistep travel speed forecasting. To make multi-time-step travel time prediction for route buses, Petersen et al. [27] proposed a convolutional long short-term memory (LSTM) neural network which used a convolutional layer to capture the spatial correlations between different route segments and used the LSTM layer to capture temporal travel time pattern. Jenelius and Koutsopoulos [28] proposed a multivariate probabilistic principal component analysis (PPCA) model which can capture spatiotemporal correlations from historical data to predict urban network travel time. They tested the PPCA model under different penetration rates and applied an EM algorithm to deal with missing data. However, the lowest penetration rate in their study was 55% which can be hardly achieved in the real world. Most researchers only focused on improving accuracy through the spatiotemporal correlation matrix because accuracy is the key index to evaluate a model. Except for the accuracy, coverage rate of a model is also critical for its application in the real world. In this study, a non-parametric model based on the spatiotemporal relationship between vehicles on the target link and vehicles in the crossing direction was proposed to enhance the coverage rate when the penetration rate is low.

In addition, most researchers chose to neglect the influence of traffic signals for the sake of simplicity when extending study sites from freeways or arterials to urban networks. For instance, Fusco et al. [22] touched upon the connection between traffic signals and individual speeds by considering the distribution of individual speeds but did not consider their overall influence. Feng et al. [29] developed a Bayesian method to determine actual traffic conditions in real-time based on synthetic GPS data and a signal timing that is known and static. The main uncertainty for urban link travel time prediction comes from the delay due to traffic signals. In this study, the signal timing was assumed to be static but unknown for simplicity because we don’t have traffic signal data in real cases so far in Japan. To maintain the accuracy of the proposed model, the influence of traffic signal was considered, and signal timing was estimated using data from vehicles in the target link and vehicles in the crossing direction.

## 3. Methodology

### 3.1. Descriptions of the Proposed Model

**Figure 1:**
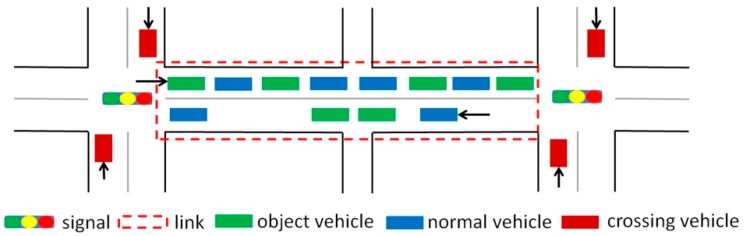
Vehicles at a regular intersection (where the penetration rate is 50%).

Urban link travel time is influenced by many factors such as signal timing, overtaking behavior, and turning at intersections. In this study, to capture the influence of traffic signals, a link is defined as a segment of the road which is separated by two adjacent signalized intersections. Many researchers have demonstrated that different turning movements experience different delays at signalized intersections and exhibit significantly different distributions [6]. For simplicity, attention was focused on predicting the travel time for vehicles going straight because vehicles turning left or right from the opposing direction must give way to vehicles going straight, which usually form the majority of the total traffic [29]. Moreover, in this study, it was assumed that there is a right-turn lane (in the case of Japan) so vehicles going straight were not influenced by vehicles with other turning choices. Because vehicles going straight have priority when going through non-signalized intersections, roads separated by non-signalized intersections were treated as one link with signalized intersections at its endpoints. The link travel time consists of both the time taken to traverse the link and the stopping time due to the traffic and the traffic signal at the downstream intersection. Hence, the exit time of a link was used as the time stamp. For clarity, the main terms in this study are defined in Table 1 and illustrated in Figure 1.

There are two main applications of the proposed model in the real world: travel time reliability analysis and reliable route searching. The prediction in the proposed model is represented by a distribution instead of the weighted summation. Travel time distribution can provide more information than the weighted summation such as the travel time variability which plays an important role in travel time reliability measurements. The proposed model can make dynamic link travel time predictions in the form of distribution and these predictions can be used as the input for reliability analysis models such as the mean-variance model [30,31]. Li et al. [31] pointed out that risk-averse travelers are willing to pay for the reduction in travel time variability rather than travel time savings. Some of them prefer the more reliable route, even though the expected travel time is higher in comparison to other routes with shorter expected travel time and higher uncertainty. Chen et al. [32] proposed a two-stage reliable path-finding algorithm and compared it with other algorithms on the urban network in Wuhan, China. They used the link travel time distributions which were estimated by existing data from a floating-car system as the input of the reliable path-finding algorithms. Because our proposed model could achieve a high coverage rate on urban networks when the penetration rate is low in the real world, its predictions of link travel time distributions can be used for real-time reliable route searching by different reliable path-finding algorithms [32]. When generating the route travel time, both link travel time and travel time covariance are summed up. In this study, the focus was limited on the link travel time prediction, so the travel time covariance estimation remains as future work.

### 3.2. Details of the Proposed Model

**Figure 2:**
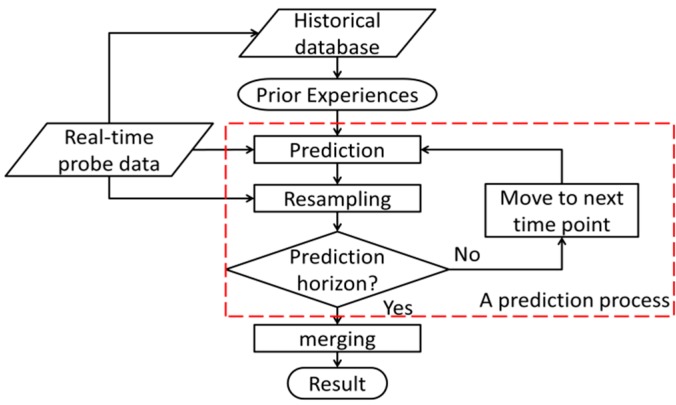
Framework of the proposed model.

The framework of our proposed model is shown in Figure 2. The proposed model is based on the prediction process consisting of prediction and resampling. According to Bucknell and Herrera [5], there is not much difference in prediction when the time interval is shorter than 5 s. In this study, the prediction time interval was set to 5 s so as to capture the frequent changes in travel time on urban networks.

The proposed model can make successive predictions as long as the computation resources are available, which means it is able to make predictions for several minutes or even hours later. However, to reflect the influence of signal timing, the prediction period is based on the average length of the green-light signal phase. Although information about signal timing might be accessible to researchers or even the public in the future, at present, it is still difficult to access in some countries such as Japan. Several methods which use trajectory data from probe vehicles have been developed to estimate signal timing [33,34,35,36]. However, in this study, there were no trajectory data available before the vehicles stop, so a simple algorithm was developed to approximately estimate the signal timing using the data collected when vehicles exit a link. This algorithm can also be applied to estimate the actuated traffic signal by data from links with similar characteristics and traffic conditions. Details of the estimation algorithm are given in the Appendix A.

For one prediction, travel time is determined by both the first observed object vehicle’s travel time and the previous travel time prediction. Provided the prediction process starts when there is an observed data $t_{n}$ at time point *n*, the probability of travel time $t_{n+l}^{j}$ at time point *(n + l)* can be calculated by

(1) $$P\left(t_{n+l}^{j}\right)=\alpha P\left(t^{j}|t_{n+l-1}^{i},D_{n+l-1}^{n+l}\right)+\left(1-\alpha \right)P\left(t^{j}|t_{n},D_{n}^{n+l}\right)$$

where $P\left(t^{j}|t_{q},D_{q}^{p}\right)$ represents the probability of travel time $t^{j}$ at time point *p* given travel time $t_{q}$ at time point *q* (p≥q) and $D_{q}^{p}$ is the leaving time difference (LTD) between *p* and *q*. Parameter α is defined as l/(l+1), which states that the object vehicle receives more influence from a vehicle that is closer to it. Next, one of the travel time candidates at time point (*n + l*) is calculated based on the first *k* possible travel times with the biggest probability using

(2) $$t_{n+l}^{i}=\overset{k}{\underset{j}{\sum }}P\left(t_{n+l}^{j}\right)t^{j}/\overset{k}{\underset{j}{\sum }}P\left(t_{n+l}^{j}\right)+\delta$$

where δ is an error term which follows a standard Gaussian distribution. To compare the proposed method with the kNN-based model, a weighted summation of these candidates is used to represent the prediction and weight $w_{m}^{i}$ is calculated by

(3) $$w_{m}^{i}=p_{N}\left(t_{m}-t_{m}^{i}\right)$$

where $t_{m}$ is the observed travel time and $p_{N}$ is a likelihood function, which is chosen to be a standard Gaussian distribution.

Algorithm 1 was proposed for predicting the travel time. The prediction horizon is defined to be the estimated green phase length *M_G_* (*M_G_*=*Length of green phase/5*), so each prediction process may have a different length (Line 1). The prediction process starts when an object vehicle is observed (Line 2). If there is more than one object vehicle observed at the same time point, the average travel time is used because the travel times are similar within 5 s in most cases. Here, 100 candidates are generated according to probability $P\left(t^{j}|t_{n},D_{n}^{n}\right)$ with the corresponding weight $w_{n}^{i}$ (Lines 3–6). Following that, candidates at step *l* are calculated (Lines 7–12). During the prediction, if a new object vehicle is observed at time point (*n + l*), a resampling process begins, and the weights are updated (Lines 13–15). If a crossing vehicle is found and it is the first crossing vehicle in the same red phase, the travel time candidate is increased by the length of a red phase *M_R_* (*M_R_*=*Length of red phase/5*) because it is assumed that the vehicle must come to a full stop to wait for the signal at the intersection (Line 19–21). Otherwise, the lengths of the candidates remain the same as those at the earlier step if a crossing vehicle is observed (Line 16–18). Moreover, if no new object vehicle is observed, the weights remain the same as that of the preceding step (Lines 22,23).
**Algorithm 1.** Proposed Travel Time Prediction Process.1:Initialize prediction horizon $M_{G}$ using Algorithm 32:**If** at time point *n*, there is an observed travel time $t_{n}$
**then**3: **For**
*i* = 1:100 **do**4:  Generate possible candidate $t_{n}^{i}$ using $P\left(t^{j}|t_{n},D_{n}^{n}\right)$ with error term δ;5:  Calculate similarity $w_{n}^{i}$ for each candidate $t_{n}^{i}$ using (3);6: **End For**7: **For**
*l* = 1:$\;M_{G}$
**do**8:  **For**
*i* = 1:100 **do**9:   For each possible travel time $t^{j}$, calculate probability $\;P\left(t_{n+l}^{j}\right)$
10:   at time point (*n + l*) using (1);11:   Calculate travel time candidate $t_{n+l}^{i}$ using (2);12:  **End For**13:  **If** object vehicle $t_{n+l}$ is observed at time point *(n + l)*
**then**14:   $w_{n+l}^{i}=p_{N}\left(t_{n+l}-t_{n+l}^{i}\right),\;\;i\in \left[1,100\right]$;15:   Begin the resampling process to modify the candidates.16:  **Else if** a crossing vehicle is observed at time point *(n + l)*
**then**
17:   **If** there is an observed crossing vehicle at time point *(n + l − p)*
**then**18:    $t_{n+l}^{i}=t_{n+l-p}^{i}$ (*l-p <*
$M_{R}$, p < l);19:   **Else**20:    $t_{n+l}^{i}=t_{n+l}^{i}+5M_{R},\;i\in \left[1,100\right]$;21:   **End If**22:   $w_{n+l}^{i}=w_{n+l-1}^{i},\;\;i\in \left[1,100\right]$;23:  **Else**, $w_{n+l}^{i}=w_{n+l-1}^{i},\;\;i\in \left[1,100\right]$;24:  **End If**25: **End For**26:**End If**

If there is no observed object vehicle, Algorithm 1 cannot be applied, and this situation often happens because of the low penetration rate. To address this situation, an object vehicle’s travel time can be estimated using the travel time distribution under the corresponding exit time difference (ETD) $d_{n}$. The ETD is defined as the difference between the exit time of an object vehicle and the last observed crossing vehicle that goes through the intersection before it.

To be specific, the 100 candidates are generated according to the conditional probability $P\left(t|d_{n}\right)$. An error term δ is also added to reflect some unexpected situations. The average travel time of the 100 candidates is used as the object vehicle’s travel time $t_{n}$. Given $t_{n}$ and 100 candidates at time point *n*, Algorithm 1 can be applied.

The prediction process tends to decrease the travel time because, in the green phase, the queue is decreasing. Although the travel time might be long at the beginning, it will soon drop to a normal level after several prediction steps. However, when congestion or accidents happen, the queue exists for longer, so the prediction is shorter than the real travel time. The resampling process relieves this problem by replacing candidates with smaller weights with candidates with bigger weights, just as in the conventional PF model. During the prediction process, if a new object vehicle is observed, it will be used to resample candidates at the current step. For example, if the real-time traffic condition is congested, the short travel-time candidates will be replaced by long travel-time candidates. However, this method lacks diversity because it resamples from existing candidates. To improve the diversity, candidates derived from probability $P\left(t|d_{n}\right)$ are introduced.

Algorithm 2 was proposed for resampling samples. Here, the resampling rate is set to 50%. Providing the resampling process starts at time point *m*$\;\left(n<m\leq n+M_{G}\right)$, the 50 candidates with the lowest weights are removed (Lines 1–3). If the ETD is less than the length of the green phase, another 100 candidates are selected according to conditional probability $P\left(t|d_{m}\right)$ and their weights are calculated by (3) (Lines 4–6). Then, new candidates whose weights are bigger than $w_{m}^{i}\;\left(i=50\right)$ are added to $\left\{t_{m}^{i}\right\}\;\left(i\in \left[1,50\right]\right)$ (Lines 7–14). If the size of the new candidate set is larger than 100, the candidates with the smallest weight are removed (Line 15–17). Otherwise, candidates are copied randomly until the size increases to 100 (Lines 18–21).
**Algorithm 2** Resampling.1:**If** at time point *m*, an object vehicle data $t_{m}$ is observed **then**2: Sort candidates according to their weight in decreasing order, 3: and remove the later 50 candidates;4: **If**
$d_{m}<M_{G}$ at time point *m*
**then**5:  **For**
*j* = 1:100 **do**6:   Select $t_{m}^{j}$ according to $P\left(t|d_{m}\right)$, calculate weight $w_{m}^{j}$ using (3);7:   **If**
$w_{m}^{i}<w_{m}^{j}\;\left(i=50\right)$
**then**
8:    $t_{m}^{k}=t_{m}^{j},\;w_{m}^{k}=w_{m}^{j}$ (*k* = 1…*K*);9:   **End If**10:  **End For**11:  Combine $\left\{t_{m}^{1},t_{m}^{2},t_{m}^{3}\ldots t_{m}^{50}\right\}$ with $\left\{t_{m}^{1},t_{m}^{2},t_{m}^{3}\ldots t_{m}^{K}\right\}$
12:  and sort candidates according to weight in decreasing order;13: **Else**
*K* = 0;14: **End If**15: **If** 50 + *K* > 100 **then**
16:  Remove the later (*K* − 50) candidates;17: **End If**18: **If** 50 + *K* < 100 **then**19:  Select (50−*K*) candidates randomly from $\left\{t_{m}^{1},t_{m}^{2}\ldots t_{m}^{50+K}\right\}$
20:  according to their weight and add them to $\left\{t_{m}^{1},t_{m}^{2}\ldots t_{m}^{50+K}\right\}$;21: **End If**22:**End If**

Because a prediction starts whenever there is an observed object vehicle or an estimated one and the process continues until it reaches the prediction horizon, there might be several predictions for the same time point. Therefore, it is necessary to merge these predictions into one. The candidates from each prediction are determined. The number of candidates is proportional to the duration between the starting and merging time points. The weight of each selected candidate is normalized, and then either a weighted summation or the candidate with the maximum weight is used to compare with the prediction results of the kNN-based and the PF-based models.

## 4. Data Process

### 4.1. Data Descriptions

In this study, models have to be tested under different penetration rates to show the influence of the penetration rate on the coverage rate. Since the probe data are usually collected from special vehicles such as taxis and buses, the penetration rate of real-world data is at a low level. Therefore, the simulation data are required to obtain traffic data at different penetration rates. VISSIM has been proven to be an effective simulation model to reproduce real-world traffic flow under different traffic conditions at both microscopic and macroscopic levels because it applies a psycho-physical car-following model that can adapt different driving behaviors [37,38]. In addition, VISSIM allows users to adjust model parameters so that it can reflect the traffic condition for a particular real-world case [37]. However, since this study focused on enhancing the coverage rate instead of improving the prediction accuracy on a certain link, default settings for the car-following model and vehicle delivery were used.

The traffic simulation was constructed using VISSIM (version 7.0) at a normal cross intersection with four 200-m-long links and attention was focused on only one link, called the target link. For simplicity, vehicles only moved straight through the intersection, so there was only one lane for each link. There was no non-signalized intersection within a link. The speed when vehicles enter the link varied from 10 to 50 km/h randomly. As for the traffic volume of the target link, it was 200, 600, and 100 vehicles/h in the first, second, and third 10 min respectively, and this pattern was repeated every 30 min. For the other three links, the traffic volume was fixed at 200 vehicles/h. The signal pattern was set to have two phases without the all-red phase, and the length of each phase was 60 s.

**Figure 3:**
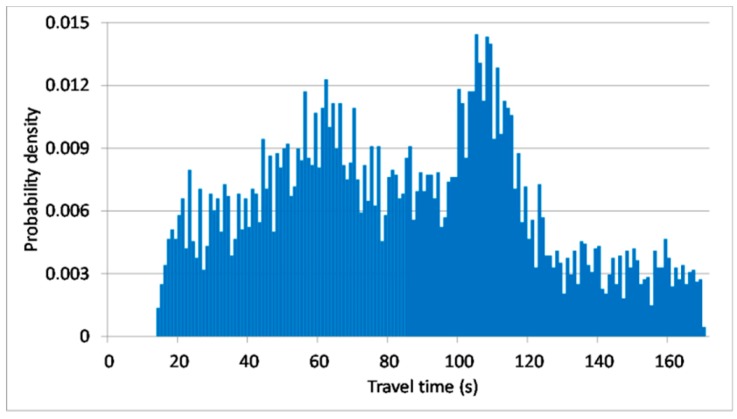
Distribution of simulation travel times.

To obtain sufficient data, the simulation was repeated 30 times. One trial was used as the test data and the remaining 29 simulations made up the historical database. The number of data in the testing database was 295, whereas the number of data in the historical database was 8568. The average link travel time in the testing database was 90.5 s, whereas in the historical database, it was 85.7 s. The distribution of the target link’s travel times is illustrated in Figure 3.

### 4.2. Relationships between Individual Vehicles

**Figure 4:**
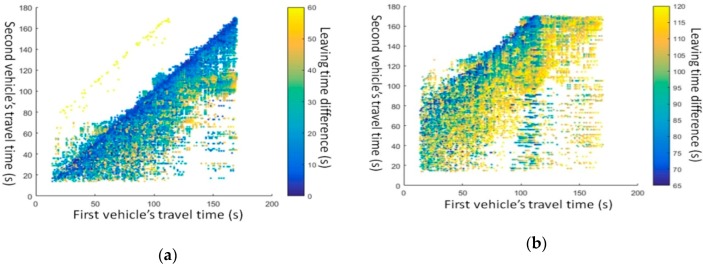
Relationship between the travel times of two object vehicles: (**a**) LTD within 60 s; (**b**) LTD more than 60 s.

The prediction process of our proposed model was based on the spatiotemporal relationship between individual vehicles. Bayes’ theorem was introduced to describe the interactions among vehicles and reflect the influence of traffic signals. Lu et al. [6] pointed out that travel time concerning signalized intersections might follow various distributions. Therefore, in this study, no specific distribution was employed. $P\left(t^{j}|t_{q},D_{q}^{p}\right)$ was derived from the relationship between the travel times for two object vehicles, while $P\left(t|d_{n}\right)$ was derived from the relationship between the ETD and the object vehicle’s travel time. The relationship between the travel times for two object vehicles is presented in Figure 4.

The horizontal axis represents the travel time of the first vehicle, whereas the vertical axis represents the travel time of the second vehicle. The color indicates the difference between the times when the two vehicles leave the link, and each dot represents a pair of travel times with different LTDs. In Figure 4a, the diagonal consists of pairs (dark blue dots) that are close to each other, which means that travel times were similar for small LTD. Most pairs are distributed under the diagonal, which means that, after the signal turned green, vehicles that arrived later had a higher probability of going through the intersection without delay or needing to stop. There is a line of yellow dots parallel with the diagonal (with a vertical axis intercept of 60 s). These dots represent two vehicles that were suddenly separated by the red signal. In Figure 4b, most pairs are distributed above the diagonal because the second vehicle was separated from the first vehicle by a red signal and was likely to stop at the intersection (the LTD ranges from 60 to 120 s). Consequently, if the first vehicle’s travel time $t_{first\;}$ and the LTD $D_{first}^{second}$ are known, the probability of travel time of the second vehicle $P\left(t|t_{first},D_{first}^{second}\right)$ can be inferred by Bayes’ theorem.

**Figure 5:**
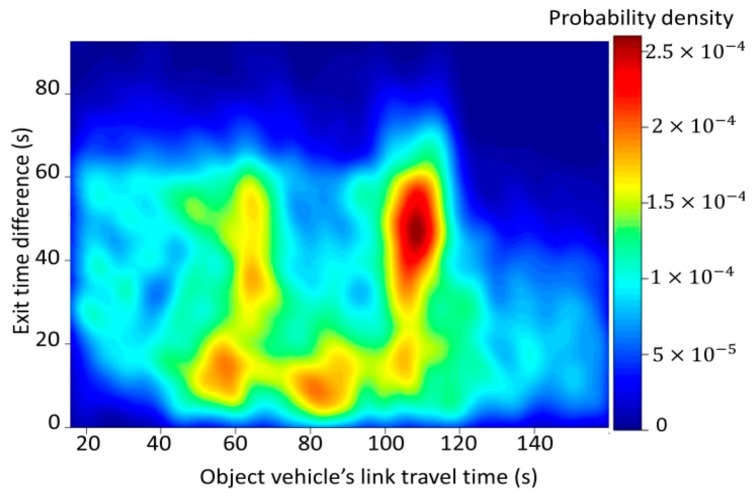
Relationship between object vehicles and crossing vehicles.

The relationship between the ETD and the object vehicle’s travel time is presented in Figure 5. When the ETD was low (e.g., less than 20 s), the travel time tended to be longer (e.g., more than 60 s) because if vehicles go through the intersection right after the signal turns green, they probably have a stop at that intersection. When the ETD increased, the probability density of short travel time increased because if vehicles go through the link during the middle or end of the green phase, they are more likely to go through the intersection without a stop. The two peaks for the long travel times when the ETD is relatively large represent congestion in which the vehicles must stop at least once at the intersection. Consequently, if the ETD is known, the probability of travel time of the object vehicle $P\left(t|d_{n}\right)$ can be inferred by Bayes’ theorem.

## 5. Experiments

### 5.1. Models for Comparison

Two non-parametric models based-on the kNN and the PF approaches were used to compare with the proposed model. For the kNN-based model, it selected time-sequential samples $\left\{x_{m}^{\left(i\right)},t_{m+l}^{\left(i\right)}\right\}\;\left(x_{m}^{\left(i\right)}=\left[t_{m-n}^{\left(i\right)},t_{m-n+1}^{\left(i\right)},\ldots ,t_{m}^{\left(i\right)}\right]\right)$ at different time points which were indicated by *m* from historical data. Here, *n* is the length of the sample and samples can be collected from different days. To predict the travel time, historical samples were compared with the current traffic condition which is measured by $x_{c}=\left[t_{c-n},t_{c-n+1},\ldots ,t_{c}\right]$. Each sample has a weight $w_{m}^{\left(i\right)}$ that represents its similarity with the current traffic condition. There are several ways to calculate the weight by selecting the distance metric, but Robinson and Polak [39] concluded that the kNN-based model is not sensitive to the distance metric. In this study, the Euclidean distance is used to calculate the weight of sample *i* as follows:

(4) $$w_{m}^{\left(i\right)}=1/\sqrt{\left(x_{c}-x_{m}^{\left(i\right)}\right){\left(x_{c}-x_{m}^{\left(i\right)}\right)}^{T}}$$

The kNN-based model can use the first *k* samples with the biggest weights to make the prediction at *l* step forward until the prediction horizon *L* (*n + l < L*) as long as there are corresponding samples, as follows:

(5) $$t_{c+l}=\overset{k}{\underset{i=1}{\sum }}w_{m}^{\left(i\right)}\cdot t_{m+l}^{\left(i\right)}/\overset{k}{\underset{i=1}{\sum }}w_{m}^{\left(i\right)}$$

Unlike the kNN-based model, the PF-based model did not traverse all the historical data. It randomly selected *N* candidates $\left\{x_{m}^{\left(i\right)},t_{m+1}^{\left(i\right)}\right\}$ and resampled part of them using the sampling importance resampling (SIR) filter to solve the problem of degeneracy [7]. According to the SIR, candidates were resampled based on their weight as follows:

(6) $$w_{m}^{\left(i\right)}\propto w_{m-1}^{\left(i\right)}\cdot \frac{p(x_{c}|t_{m}^{\left(i\right)})p(t_{m}^{\left(i\right)}|t_{m-1}^{\left(i\right)})}{q(t_{m}^{\left(i\right)}|t_{m-1}^{\left(i\right)},x_{c})}$$

In the SIR, the importance density $q(t_{m}^{\left(i\right)}|t_{m-1}^{\left(i\right)},x_{c})$ was assumed to have the same value as the transitional prior pdf $p(t_{m}^{\left(i\right)}|t_{m-1}^{\left(i\right)})$, so (6) can be simplified as (7) where $p\left(x_{c}-x_{m}^{\left(i\right)}\right)$ represents the similarity between the candidate and the current traffic condition. There are also several ways to calculate the weight, but the same calculation as (4) was used for the PF-based model in this study. Readers can refer to [7] for more details about the PF in travel time prediction.

(7) $$w_{m}^{\left(i\right)}\propto p\left(x_{c}|t_{m}^{\left(i\right)}\right)=p\left(x_{c}-x_{m}^{\left(i\right)}\right)$$

The PF-based model can make predictions successively by shifting the time window one step forward at each step until the prediction horizon *L*, as follows:

(8) $$t_{c+1}=\overset{N}{\underset{i=1}{\sum }}w_{m}^{\left(i\right)}\cdot t_{m+1}^{\left(i\right)}/\overset{N}{\underset{i=1}{\sum }}w_{m}^{\left(i\right)}$$

**Figure 6:**
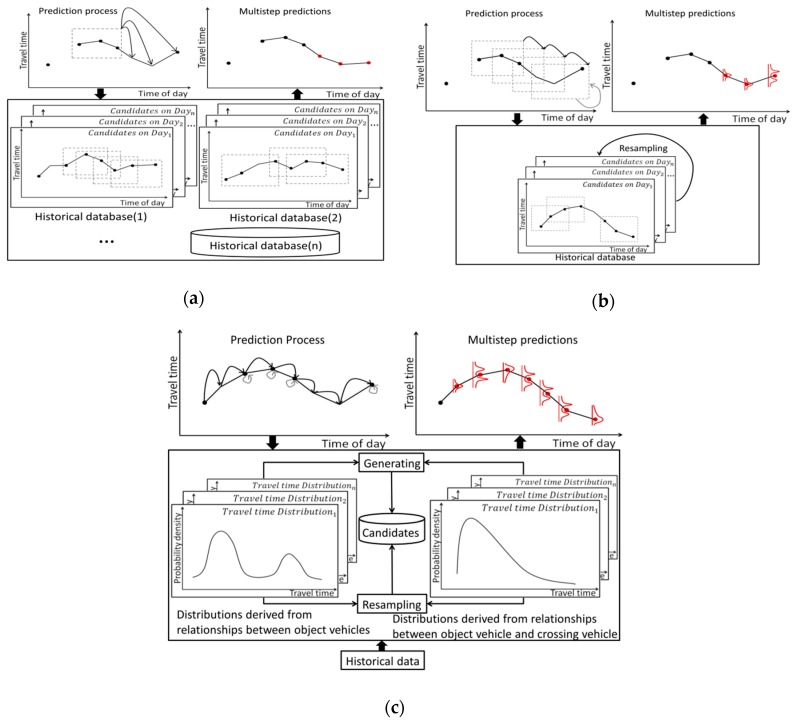
Models used in this study: (**a**) kNN-based model; (**b**) PF-based model; (**c**) Proposed model.

As for the historical database, the PF-based model only needs one database once the length of the candidate is decided because its prediction step is identical. In contrast, the kNN-based model needs several historical databases according to the prediction horizon. Process of the three models is illustrated in Figure 6, where the black dots represent the observed travel time of an object vehicle, whereas the red dots represent the model predictions.

In the proposed model, the historical data provide the prior travel time distributions based on the spatiotemporal relationships between object vehicles and the spatiotemporal relationships between object vehicles and crossing vehicles. Therefore, a prediction can be made whenever there is an observed data sample (i.e., an object or crossing vehicle). In contrast, the kNN-based and the PF-based models can only make a prediction when there are continuously observed object vehicle data. Because of the use of information from crossing vehicles to make predictions, the proposed model is expected to achieve a higher coverage rate than the other two models. As mentioned before, to make a prediction at one time point, there might be several prediction processes in the proposed model, depending on the number of observed vehicles during the prediction period. Therefore, it was difficult to calculate the computation cost for one prediction in the proposed model because it varied from case to case. However, the proposed model was expected to have higher computation cost than the kNN-based and the PF-based models which only have one prediction process for one prediction.

### 5.2. Experiment Settings

One-third of the testing data collected from the target link was selected randomly as travel times for prediction (target data), whereas the remaining two-thirds were treated as travel time that can be observed during the prediction (observed data). Then, some observed data and data from crossing vehicles (crossing vehicle data), were removed randomly to simulate the situation under different penetration rates. In the kNN- and the PF-based models, historical samples with a similar pattern of the current traffic condition were used to predict the travel time for a certain link. Because different links have different characteristics, historical samples from one link can only be used to predict the travel time for that link. That is the reason why kNN-based and PF-based models cannot use the crossing vehicle data to predict the travel time for the target link. However, the correlation between adjacent links can help improve the accuracy of travel time prediction so many researchers applied the crossing vehicle data to the link travel time prediction as introduced in Section 2. Because the accuracy improvement is not the interest of this paper, the crossing vehicle data were not used in the kNN-based and the PF-based models in this study. On the other hand, the proposed model without considering crossing vehicles, namely proposed model β (*PM_*β*)*, should be tested to evaluate the influence of crossing vehicles because the travel time of the target link can be predicted directly from the crossing vehicle data in the proposed model.

As for the parameter calibration in the kNN-based model, Robinson and Polak [39] pointed out that attention should be paid to determining the optimal value of *k* which depends on the database size. In their research, no matter how the database size changed, the optimal value of *k* was less than 300 and the difference between the mean absolute percentage error (MAPE) with a different value of *k* within that range was within 5%. A similar pattern of the relationship between the MAPE and the value of *k* as that in Robinson and Polak’s [39] research can be found in other kNN-based studies [9,21] and the difference between the MAPE with a different value of *k* in these studies was also within 5%. Although the value of *k* affects the accuracy of the kNN-based model, it is reasonable to fix the value of *k* without calibration because a 5% difference is at an acceptable level when the main concern of this study is not improving the accuracy. Consequently, the value of *k* in (5) was set as 4 and the sample length *n* was set as 2 to ensure the database size is large enough in advance.

For the parameter calibration in the PF-based model, Chen and Rakha’s [7] work showed that although the number of candidates and the value of the resampling rate influenced the accuracy of the PF-based model, the difference in the MAPE was also within 5%. For the same reason, the number of candidates and the value of the resampling rate were fixed in advance without calibration. The number of candidates *N* in (8) was set as 100. The resampling rate was set as 50%, which means that the latter 50 candidates were replaced by the former 50 candidates according to their weight.

This study aims to maintain the accuracy of the proposed model at the same level as that of the kNN-based and the PF-based models, so some parameters in the proposed model were set as the same value as those in the two comparison models in advance. The value of *k* in (2) was set as 4, the number of candidates was set as 100, and the resampling rate was set as 50%. The discussion of α is out of the scale of this study so it remains as future work. In addition, the time interval and prediction horizon for the three models were the same.

### 5.3. Results and Discussion

**Figure 7:**
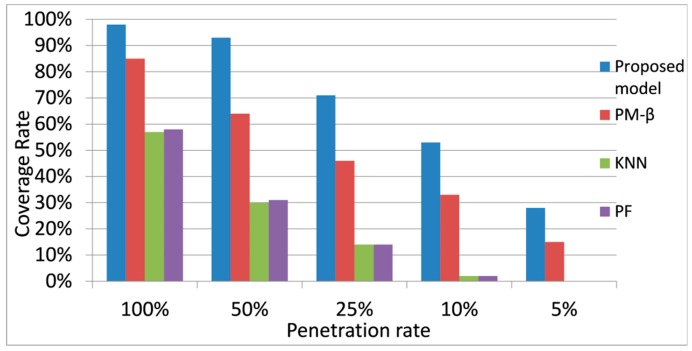
Coverage rate under different penetration rates.

Although the number of target data was the same under different penetration rates, not all of them can be predicted. The proportion of target data that can be predicted was referred to as coverage rate. Coverage rate under different penetration rates for different models is shown in Figure 7. Coverage rate for each model shrank when the penetration rate decreased, but the coverage rate of the proposed model was always higher than that of other models. This coverage rate was also stable if the penetration rate was more than 50%. Even when the penetration rate was less than 50%, the proposed model could cover over half of the points in time if the penetration rate was no less than 10%.

The same historical database was used for different penetration rates because it was assumed that sufficient data can be collected, regardless of the penetration rate, if the period of data collection was reasonably long. Consequently, the spatiotemporal relationships and signal timing estimation were the same for different penetration rates. The estimation of the green phase was 65 s with a 5-s variance, whereas the estimation of red phase was 55 s with a 5-s variance. The estimation was comparatively close to the setting, which was 60 s for both phases. 

Two indices were used to measure the accuracy of the proposed model. One was the MAPE and the other was the root mean squared error (RMSE), defined as follows:

(9) $$MAPE=\frac{1}{N}\overset{N}{\underset{i}{\sum }}\frac{\left|t_{i}-{\hat{t}}_{i}\right|}{t_{i}}$$

(10) $$RMSE=\sqrt{\frac{1}{N}\overset{N}{\underset{i}{\sum }}{\left(t_{i}-{\hat{t}}_{i}\right)}^{2}}$$

where *N* is the total number of predictions. At time point *i*, $t_{i}$ is the true value of the travel time, whereas ${\hat{t}}_{i}$ is its prediction.

The accuracy of the proposed model under different penetration rates is shown in Table 2.

The accuracy deteriorated slightly when the penetration rate decreased, but it remained at a stable level. If the average value of travel time in the historical database was used as the prediction, the accuracy increased when the penetration rate decreased. This is because, when the penetration rate decreased, the traffic conditions where predictions can be made were probably in congestion, and the average travel times in the historical database were relatively long. Nevertheless, the proposed model had higher accuracy than just using the average value under different penetration rates.

Because the coverage rate of each model under different penetration rates varied and the proposed model can make predictions at more points in time, it was unreasonable to compare the MAPE or RMSE directly. Therefore, the following adjusted measurements of accuracy were used in this study:

(11) $$Model-diff.MAPE=\frac{1}{N_{a}}\left(\overset{N_{a}}{\underset{i}{\sum }}\frac{\left|t_{i}-{\hat{t}}_{i}^{PM}\right|}{t_{i}}-\overset{N_{a}}{\underset{i}{\sum }}\frac{\left|t_{i}-{\hat{t}}_{i}^{M}\right|}{t_{i}}\right)$$

(12) $$Model-diff.RMSE=\sqrt{\frac{1}{N_{a}}\overset{N_{a}}{\underset{i}{\sum }}{\left(t_{i}-{\hat{t}}_{i}^{PM}\right)}^{2}}-\sqrt{\frac{1}{N_{a}}\overset{N_{a}}{\underset{i}{\sum }}{\left(t_{i}-{\hat{t}}_{i}^{M}\right)}^{2}}$$

where *Model* represents the name of the comparison method and $N_{a}$ is the total number of predictions it can make. At time point *i*, ${\hat{t}}_{i}^{PM}$ is the prediction of the proposed model, and ${\hat{t}}_{i}^{M}$ is the prediction of the comparison method.

The accuracy for comparing methods under different penetration rates is shown in Table 3. Because the coverage rates of the kNN-based and the PF-based models were remarkably low when the penetration rate was no more than 10%, results are not shown. The kNN-based model outperformed the proposed model when the penetration rate was remarkably high, but this advantage was not obvious. When the penetration rate decreased, the accuracy of the proposed model became better than that of the kNN-based and the PF-based models. Considering the coverage rate, the performance of the proposed model was better than the kNN-based and the PF-based models under different penetration rates. As for *PM_*β, there was little difference between the proposed model and *PM_*β, so crossing vehicles did not influence the accuracy when data from the object vehicle were plentiful. Nevertheless, if the object vehicle was unavailable, crossing vehicles could provide information to make predictions at a similar level of accuracy, so it is necessary to consider crossing vehicles to achieve a higher coverage rate.

**Figure 8:**
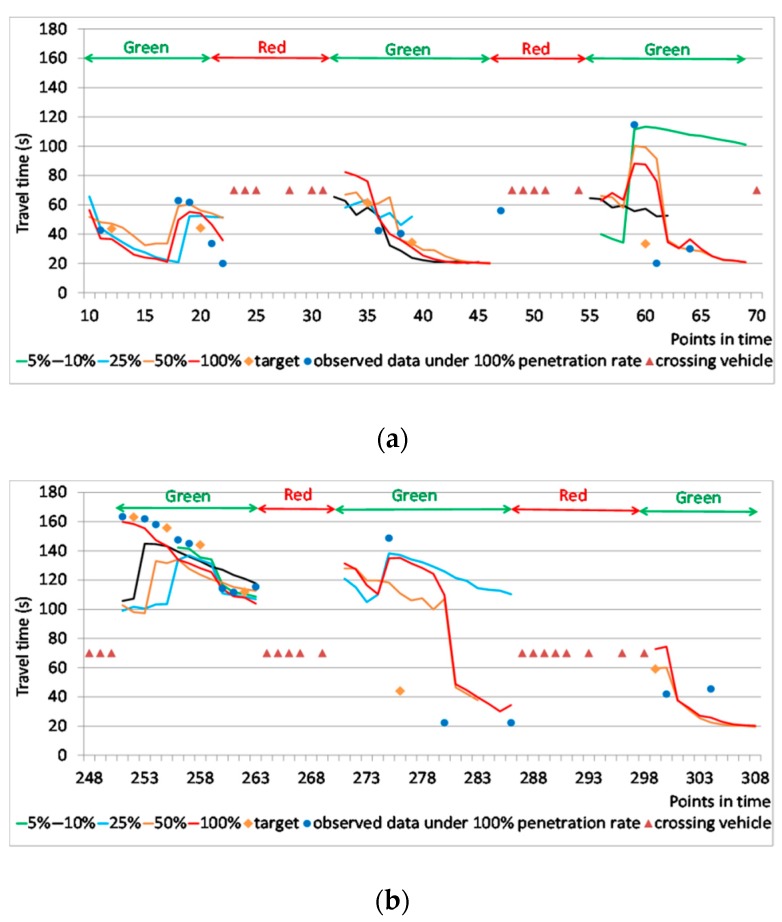
Predictions of the proposed model under different penetration rates: (**a**) Time points from 10 to 70; (**b**) Time points from 248 to 308.

Some examples of predictions by the proposed model under different penetration rates are shown in Figure 8. The orange dots (target) represent the travel times for prediction, whereas the blue dots represent observed data during prediction under a 100% penetration rate. For simplicity, the travel time of the crossing vehicle was changed to 70 s because the value of travel time does not affect the prediction. Because there is no vehicle passing through the intersection when the signal is red, there is no prediction during the red phase where the crossing vehicles appear successively. If the prediction during the red phase is needed, for example, for the route choice task, the prediction at the beginning of the following green phase can be used because it includes the stopping time at the intersection.

The proposed model could make predictions under most traffic conditions if the penetration rate was no less than 25%, but it might not be useful under unsaturated traffic conditions if the penetration rate was lower than 25%. For example, when congestion was disappearing, the proposed model could not work at the low penetration rate in Figure 8b. The proposed model could reflect the fact that link travel time decreases after the signal turns green and the increase in travel time at the beginning of green phase due to the stop at the intersection, especially under unsaturated traffic condition, as shown in Figure 8a. When the penetration rate was high, the proposed model could trace the change in travel time resulting from congestion using its resampling process. However, the proposed model could not react to the sudden change in traffic conditions promptly unless there were data observed after the change. For example, in Figure 8b, from time points 273 to 283, the congestion started to disappear, but the proposed model did not reflect the drop in travel time until new data were observed. However, the resampling process sometimes made the prediction overreact to some unexpected change in travel time, as shown in time points 55 to 60 in Figure 8a.

## 6. Conclusions and Future Work

Generally speaking, the proposed model had a higher coverage rate and stable performance under different penetration rates because it used the information from both object vehicles and crossing vehicles as well as a resampling process to trace the change in travel time due to unexpected events. In this study, although the crossing vehicle data did not contribute to the accuracy improvement, they can make travel time prediction at the same level as using the data from the target link. Therefore, the proposed model can significantly enhance the coverage rate when applying to the urban networks where the penetration rate of probe vehicles is low. Since the distribution of probe vehicles is not uniform in the real world, most researches have difficulty in practical application because they have limited coverage rate. However, the proposed method can predict link travel time in the form of distribution on the whole urban network for searching the most reliable route wherever the penetration rate of probe vehicles is low. Furthermore, the travel time distribution can provide more information than a weighted summation in practical applications, such as travel time reliability analysis.

There is still much room for improvement in the proposed model. In the future, the focus will be concentrated on the following tasks: (i) consider the intersection control by including different turning choices, (ii) calibrate parameters of the proposed model for higher accuracy, (iii) reduce the computation cost, (iv) consider the actuated traffic signal, and (v) test the proposed model in the real-world network and realize applications, such as travel time reliability analysis and reliable route searching.

## Figures and Tables

**Table 1:** Definition of terms related to vehicles.

Term	Definition
Object vehicle	Probe vehicle that travels straight through the downstream signalized intersection
Normal vehicle	Vehicle that cannot send probe data
Crossing vehicle	Probe vehicle traveling in the crossing direction that goes through the same downstream signalized intersection
Penetration rate	The ratio of probe vehicles to all vehicles
Coverage rate	The proportion of travel time that can be predicted

**Table 2:** Accuracy for the proposed model under different penetration rates.

Penetration Rate (%)	100	50	25	10	5
Proposed model MAPE (%)	19.3	25.6	26.2	26.5	33.8
Proposed model RMSE	19.7	24.4	29.2	27.3	30.7
Average value MAPE (%)	71.9	65.5	57.5	58.9	54.0
Average value RMSE	43.1	42.6	44.3	39.8	34.2

**Table 3:** Accuracy for comparison methods under different penetration rates.

Penetration Rate (%)	100	50	25	10	5
kNN-diff.MAPE (%)	3.2	−1.0	−8.0	−	−
kNN-diff.RMSE	2.0	−1.0	−5.0	−	−
PF-diff.MAPE (%)	−12	−12	−27	−	−
PF-diff.RMSE	−9.0	−9.0	−15	−	−
*PM_*β-diff.MAPE (%)	0.0	2.0	0.0	−1.0	−1.0
*PM_*β-diff.RMSE	−3.0	1.0	2.0	0.0	−6.0

## Appendix A

To build a relatively sufficient historical database, it is assumed that the signal pattern remains the same during the same period of day. In this algorithm, one signal cycle is divided into two phases—the green phase when the object vehicle can move straight through the intersection, and the red phase when the crossing vehicle can move through the intersection. There are two problems in estimation. One is the errors; for example, there might be an object vehicle data appearing in a stream of crossing vehicle data, which is physically impossible. The other one is an overestimation of the signal phase due to the lack of data.

Algorithm 3 was proposed for estimating the signal timing. The historical data of object vehicles and crossing vehicles at the intersection from the same day are sorted as a time sequence. If there are object vehicles going through the intersection, the signal is green; otherwise, the signal is red, and vice versa (Line 1–6 and 13–17). To address the first problem, the phase is changed when its length is under 40 s (Line 7–11 and 18–22). Because traffic signals change successively, the phase at the beginning and end of the time period is removed (Line 12 and 23). To address the second problem, the red phases in {S2} over 80 s long are replaced by corresponding phases in {S1} (Line 24–28). Then, samples of the green phase and the red phase can be obtained from {S2}. Finally, the length of the green phase can be represented by the summation of the average length of the green phase (*AVE_green_*) between 40 s and 80 s long and its standard deviation (*SD_green_* as shown in A(1) and A(2) (Line 31–32). Similarly, the length of the red phase can be represented by the summation of the average length of the red phase (*AVE_red_*) between 40 s and 80 s long and its standard deviation (*SD_red_*) as shown in A(3) and A(4) (Line 33–34):

(A1) $$Length\;of\;\;green\;phase=AVE_{green}+\varepsilon_{green}$$

(A2) $$\mathrm{where}\;\varepsilon_{green}=\{\begin{array}{c} SD_{green},\;\;\delta \in \left(SD_{green},+\infty \right)\\ \delta ,\;\;\;\delta \in \left[-SD_{green},SD_{green}\right]\;\;\\ -SD_{green},\;\;\delta \in \left(-\infty ,-SD_{green}\right) \end{array}\;\mathrm{and}\;\delta \sim \left(0,1\right)$$

(A3) $$Length\;of\;\;red\;phase=AVE_{red}+\varepsilon_{red}$$

(A4) $$\mathrm{where}\;\varepsilon_{red}=\{\begin{array}{c} SD_{red},\;\;\delta \in \left(SD_{red},+\infty \right)\\ \delta ,\;\;\;\delta \in \left[-SD_{red},SD_{red}\right]\;\;\\ -SD_{red},\;\;\delta \in \left(-\infty ,-SD_{red}\right) \end{array}\;\mathrm{and}\;\delta \sim \left(0,1\right)$$

Here, 40 s and 80 s are used as the thresholds because in Japan, it is common for the average length of a signal phase to be 60 s with a variance of 20 s. Error term δ is added to reflect unexpected events, such as a change in signal pattern. It is assumed that δ follows a standard Gaussian distribution. Provided the historical data are abundant and precise, samples in {S1} or {S2} can be used alone without overestimating the length of the phase.

**Algorithm 3**: Signal timing estimation	
1:	**For** continuous data streams consists of object vehicle data and crossing vehicle data **do**
2:	**If** the object vehicle data appear continuously during a period **then**
3:	Record this period as the green phase
4:	Intervals between each green phase are recorded as the red phase
5:	This record is denoted as {S1}
6:	**End If**
7:	**For** all red phases in {S1} **do**
8:	**If** the length of a red phase is shorter than 40s **then**
9:	Change this red phase into the green phase
10:	**End If**
11:	**End For**
12:	Remove the first and last phases in {S1}
13:	**If** the crossing vehicle data appear continuously during a period **then**
14:	Record this period as a the red phase
15:	Intervals between each red phase are recorded as the green phase
16:	This record is denoted as {S2}
17:	**End If**
18:	**For** all green phases in {S2} **do**
19:	**If** the length of a green phase is shorter than 40s **then**
20:	Change this green phase into the red phase
21:	**End If**
22:	**End For**
23:	Remove the first and last phases in {S2}
24:	**For** all red phases in {S2} **do**
25:	**If** the length of a red phase is longer than 80 s **then**
26:	Replace this red phase in {S2} with the corresponding phase in {S1}
27:	**End If**
28:	**End For**
29:	**Return** samples of the green phase and the red phase in {S2}
30:	**End For**
31:	Calculate $AVE_{green}$ and $SD_{green}$ with samples of the green phase between 40 s and 80 s long
32:	Calculate the length of the green phase by A(1) and A(2).
33:	Calculate $AVE_{red}$ and $SD_{red}$ with samples of the red phase between 40 s and 80 s long
34:	Calculate the length of the red phase by A(3) and A(4).
